# Emerging technologies toward the integration of multiple functionalities on non-planar implantable neurophotonics probes

**DOI:** 10.1117/1.NPh.11.S1.S11514

**Published:** 2024-08-09

**Authors:** Mohammad Mohammadiaria, Marco Bianco, Antonio Balena, Maria Samuela Andriani, Cinzia Montinaro, Barbara Spagnola, Filippo Pisano, Ferruccio Pisanello, Massimo De Vittorio

**Affiliations:** a Istituto Italiano di Tecnologia, Center for Biomolecular Nanotechnologies, Arnesano, Italy; b Sorbonne University, CNRS, ENS-PSL University, Collège de France, Laboratoire Kastler Brossel, Paris, France; c Università del Salento, Dipartimento di Ingegneria Dell’Innovazione, Lecce, Italy; d RAISE Ecosystem, Genova, Italy; e Università di Padova, Dipartimento di Fisica e Astronomia “Galileo Galilei,” Padova, Italy

**Keywords:** fiber optics, multifunctional probes, electrophysiology, optogenetics, two-photon lithography

## Abstract

The continuous exchange between the neuroscience and neuroengineering communities that took place over the past decades has uncovered a multitude of technological solutions to interface with the brain. In this framework, a fascinating approach relies on the integration of multiple activation and monitoring capabilities in the same implantable neural probe to better study the multifaceted nature of neural signaling and related functions in the deep brain regions. We highlight current challenges and perspectives on technological developments that could potentially enable the integration of multiple functionalities on optical fiber-based non-planar implantable neurophotonics probes.

## 1 Introduction

Deciphering how complex neural processes are organized into neural functions that involve elaborate signal exchanges across multiple neurons has always been the mission of neuroscientific research.^1^^,^^2^ This ambitious goal has driven the neurotechnology community to provide increasingly advanced technologies so that neuroscientists can interface with the multifaceted nature of neural signals. Although the recording of electrophysiological signals has been the best channel for interfacing with the brain for decades, the advent of optical cell-specific interfacing methods has overwhelmingly pushed toward the study of optical implantable probes.^3^^–^^5^ Prominent among these are optical fibers, due to their simplicity of use, ease of implantation, low cost, and the ability to modify the employed wavelength in situ. Immediately thereafter, the union of electrophysiological and optical interface channels was proposed as the first example of a multifunctional neural interface,^6^^–^^8^ with the goal of integrating activation and recording channels on a single probe, thus expanding the possibilities of designing increasingly elaborate neuroscientific experiments. The objective of this perspective article is to identify emerging technologies that have the potential to increase the integration of electrical and electrochemical detection sites on implantable fiber-optic-based probes, toward high-density electrode integration and high scalability of the manufacturing. After a brief overview of how the key features of purely high-density electrophysiological probes and those of fiber-optic-based probes complement each other, the hallmarks of some promising technologies will be introduced, along with some limitations that, if overcome, could prospectively lead to wider and more reliable use. Although special emphasis is given here to the integration of electrophysiological and optical capabilities, it will become evident how these technologies may lead to the enrichment of other features such as drug delivery from microfluidic channels, light collection for photometry or Raman spectroscopy, and exploitation of plasmonic effects.

## 2 Electrophysiology and Optical Neural Interfaces

Building on the first pioneering experiments^9^ in the 1950s, extracellular electrophysiology can now benefit from a plethora of probe designs to adapt to the experimental requirements such as micro-electrocorticography arrays,^10^ microneedle-shaped probes,^11^ Michigan-style microelectrode arrays,^12^ and Utah arrays,^13^ to name a few, which have been employed in rodents, non-human primates, and humans. Unprecedented microelectrodes recording density^14^^–^^16^ brought closer the goal of measuring *all neurons* at the same time,^17^ which would require an electrode density of approximately $2.6\times 10^{5}\;\text{channels}/{\mathrm{mm}}^{2}$, but the consequent and inevitable shrinkage of the electrode size reflects on increased impedance, hence reducing the signal-to-noise ratio (SNR) and resilience to thermal noise.^18^ These aspects drove a complementary effort in the study of built-in preamplification/processing systems, to increase the SNR through local amplifiers such as transistors. In this respect, a particularly interesting configuration is the electrochemical field-effect transistor (eFET), which in turn includes the wide family of organic electrochemical transistors (OECT).^19^^,^^20^ Those novel probes allowed for high SNR^21^^,^^22^ and spatiotemporal resolution,^23^ as well as high-density recordings of extracellular action potentials,^24^ and could be easily functionalized for the detection of specific biochemical species.^25^ However, almost the totality of examples is limited to planar geometries, fitted for shallower cortical investigations, but not allowing for deep-brain interfacing.

Although electrophysiological interfacing with the brain remains the prevalent choice in neuroscience research,^26^ the experimental possibilities that arose after the introduction of optical neural interfacing methods^27^^–^^31^ have complementarily turned the spotlight on implantable optical devices, such as micro-LED (μLED) arrays,^32^ ridge waveguides,^33^ and fiber optics.^34^^–^^37^ Those latter allow accessing and interfacing with deep-brain regions, hardly accessible even for advanced microscopy techniques,^38^^,^^39^ while offering cell-type specificity thanks to the genetic encoding of optical actuators/reporters, as opposed to the lack of specificity of electrical measurements. Still, electrophysiological and optical techniques should be considered complementary to each other. If optogenetics allows for cell-type–specific neural stimulation, optical readout of neural activity mainly focuses on high-resolution imaging of calcium dynamics^40^ or membrane voltage,^41^ and it is far from the possibility of catching local field potentials. Furthermore, genetically encoded fluorescent reporters are still limited to a few molecular species,^42^ while the perspective of electrochemical detection performed by implantable OECT arrays could extend from neurotransmitters^43^ to gaseous species such as nitric oxide.^44^ These considerations are pushing the scientific community to strongly advance the field of multifunctional probes that combine optical and electrical access to brain signals,^7^^,^^45^^–^^47^ with the perspective of building multifunctional closed-loop systems^48^ and offering a better understanding of the complexity of neural signaling. In this framework, multimode optical fibers have seen a strong research focus thanks to the wealth of information that can be carried by the propagation of the electromagnetic field, while keeping a small implant cross-section. To add multiple functionalities together with the optical channels, the scientific community is dealing with the fact that traditional micro-electromechanical systems (MEMS) fabrication techniques^49^ may be inadequate to pattern the non-planar surface or the bulk of the core/cladding structure of optical fibers. In this respect, several promising *ad hoc* techniques have been proposed by the community to tackle, even partially, those limits and realize increasingly advanced fiber-based probes.

## 3 Advanced Micro- and Nano-Fabrication Methods for Multifunctional Fiber-Based Interfaces

### 3.1 Fiber Drawing

**Fig. 1:**
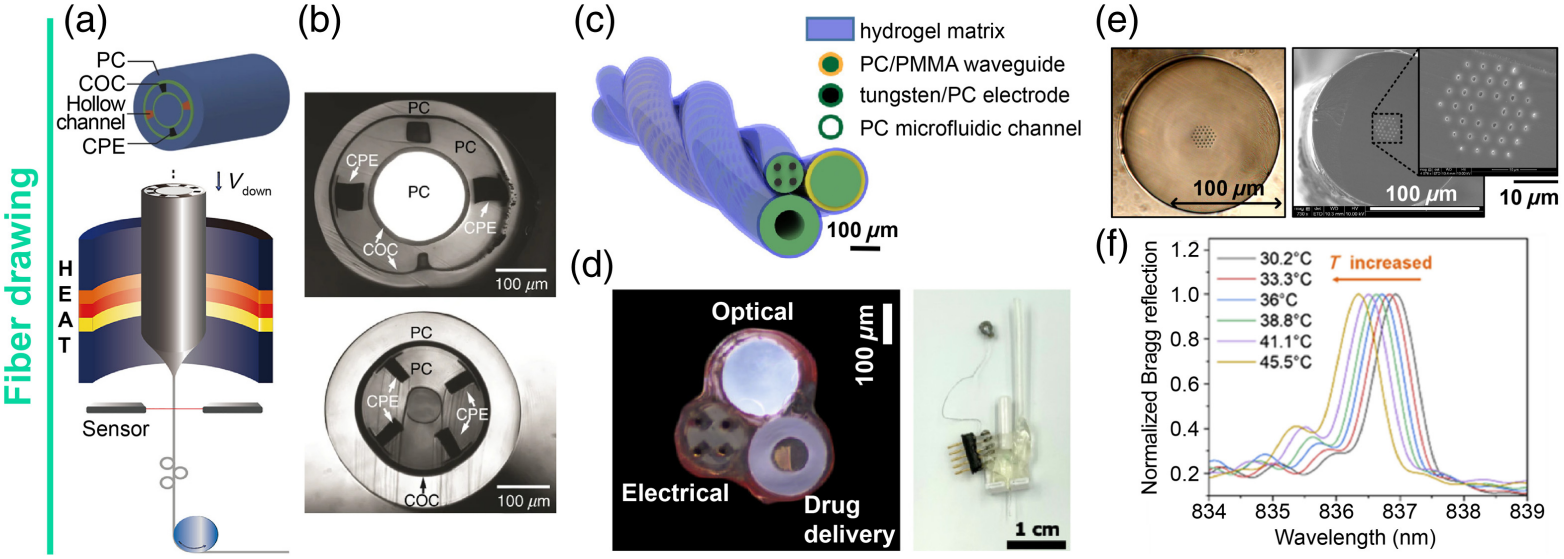
Fiber drawing. (a) Schematic representation of the thermal drawing process. Adapted from Ref. 50. (b) Cross-sectional optical images of different designs of multi-modality thermally drawn fibers. Adapted from Ref. 46. (c) Graphical representation and (d) cross-sectional optical images of a twisted polymeric multifunctional probe. Each channel is individually drawn and then assembled in the final probe. Adapted from Ref. 51. (e) Optical image and scanning electron micrograph of an FBG inscribed in a polymeric optical fiber through the phase-mask technique. Reproduced from Ref. 52. (f) Normalized Bragg reflection at different temperatures, showing a blueshift as the temperature increases. Reproduced from Ref. 53.

Among the emerging technologies conceived for the achievement of advanced fiber-based multi-functional interfaces, a notable role is played by fiber drawing, which allows for combining an optical interface, electrodes for electrophysiology, and drug delivery capillaries in a single thermally drawn polymeric optical fiber. For example, Canales et al. did extensive work^46^^,^^50^ proposing a thermal drawing process (TDP) that simultaneously combines polymers, metals, and composite materials to obtain a probe that achieved simultaneous optogenetic stimulation, long-term neural recordings, as well as drug delivery in freely moving animals [Figs. 1(a)–1(b)]. An alternative approach is to twist together multiple channels that have been previously drawn singularly, as proposed by Tabet et al.,^51^ which demonstrated simultaneous optogenetics and electrophysiology and showed cellular cargo delivery with high viability from a modular probe encased in a hydrogel matrix [Figs. 1(c)–1(d)]. In other cases, thermally drawn fibers have been combined with phase masking techniques to integrate fiber Bragg grating (FBG) sensor^52^ to add thermometric capabilities to the implantable probe, which have been used to measure the correlation between the brain and body core temperature of a rat with a <0.2°C accuracy *in vivo*, as demonstrated by Sui et al.^53^ [Figs. 1(e)–1(f)]. TDP fibers also benefit from a reduced Young’s modulus, exhibiting mechanical properties more similar to the brain tissue with respect to silicon or silica implantable devices, thus allowing for reduced tissue damage during the implantation phase. TDP allows the integration of multiple functionalities into minimally invasive probes; however, they still present some disadvantages compared with conventional fiber-optic devices: due to the higher-decibel loss characteristics of polymeric fibers with respect to conventional silica fibers, these latter are still preferred for bidirectional fiber photometry and/or Raman spectroscopy applications. In addition, the choice of the polymer employed during the drawing may constrain further micro-/nano-structuration of the probes. These include high-vacuum metal deposition or patterning processing, as well as high-temperature dewetting for metal nano-particle decoration, which represents one of the most straightforward ways toward surface plasmon resonances (SPR)- or localized SPR-based chemical sensing applications.^54^^,^^55^ We believe that upcoming technological developments in material science may offer an opportunity to surpass those drawbacks, resulting in flexible optical fibers characterized by high transmission and improved material characteristics.

### 3.2 Transfer Printing

**Fig. 2:**
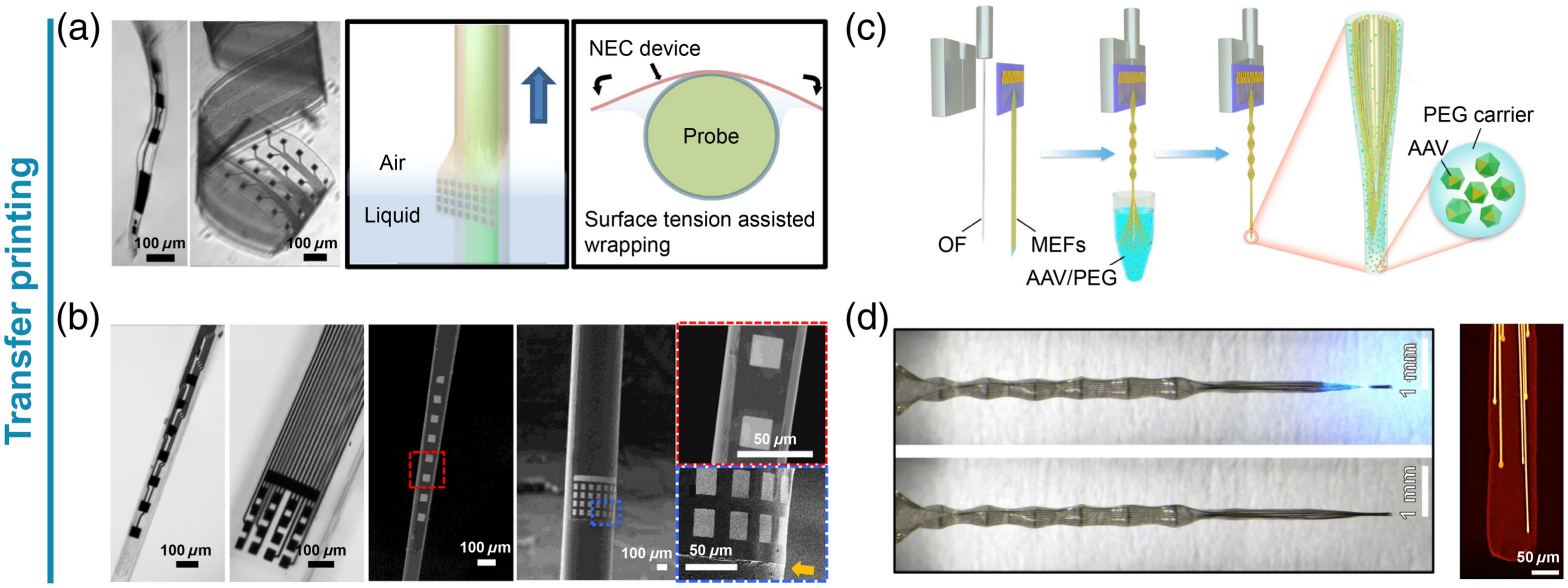
Transfer printing. (a) Optical images of released flexible microelectrode arrays and a sketch of the wrapping process facilitated by surface tension. (b) Optical images and scanning electron micrographs of multifunctional probes obtained by transfer printing. Panels (a) and (b) are reproduced from Ref. 56. (c) Schematics of the self-assembly of a flexible microelectrode array around an optical fiber. (d) Optical images of the final device, with and without blue light illumination, and a false-color micro-CT image of the probe. Panels (c) and (d) are reproduced from Ref. 57.

An alternative approach with great potential in multifunctionality integration on optical fibers is transfer printing. It may conceivably find extended adoption, especially in integrating a relatively high number of microelectrodes around a non-planar implantable probe. This method dodges the challenges of integrating multiple recording sites on a small, curved surface by fabricating relatively complex arrays through planar MEMS fabrication techniques on a flexible membrane, which is then wrapped around an existing rigid, non-planar probe. In this way, Zhao et al.^56^ enhanced different non-electrical implants with a diameter that ranged between 30 and 200  μm with electrical recording capabilities thanks to a flexible SU-8 electrode array [Figs. 2(a)–2(b)], wrapped around the probes through surface tension-assisted wrapping, also optimizing the relation between the flexible device thickness and the wrapped probe to ensure optimal wrapping. Furthermore, the membrane can be wrapped around a rigid optical fiber, to achieve optrode functionalities ready for deep-brain regions. Zou et al. further engineered the flexible wrapping realizing a viral vector-delivery optrode, by wrapping an array of flexible microelectrode filaments embedded in an adeno-associated virus (AAV) vector and poly(ethylene glycol) (PEG) matrix around an optical fiber [Figs. 2(c)–2(d)].^57^ After implantation, the PEG dissolves, releasing the AAV for localized transduction of nearby neurons. The optrodes allowed simultaneous optogenetic stimulation and multi-channel recording for three months. Aiming to a widespread utilization, it would be beneficial to optimize two main aspects of the processing: (i) being based on a sort of self-assembly mechanism, workarounds for precise relative positioning of the fiber and the flexible mesh must be implemented, since uncontrolled wrapping could result in misplacement of the active elements along the probe axis and (ii) the same mechanism may also result in sub-optimal adhesion of the mesh on the fiber, especially when extended flexible membranes are to be wrapped, thus undermining the implantation of the probe in the tissue.

### 3.3 Two-Photon Lithography

**Fig. 3:**
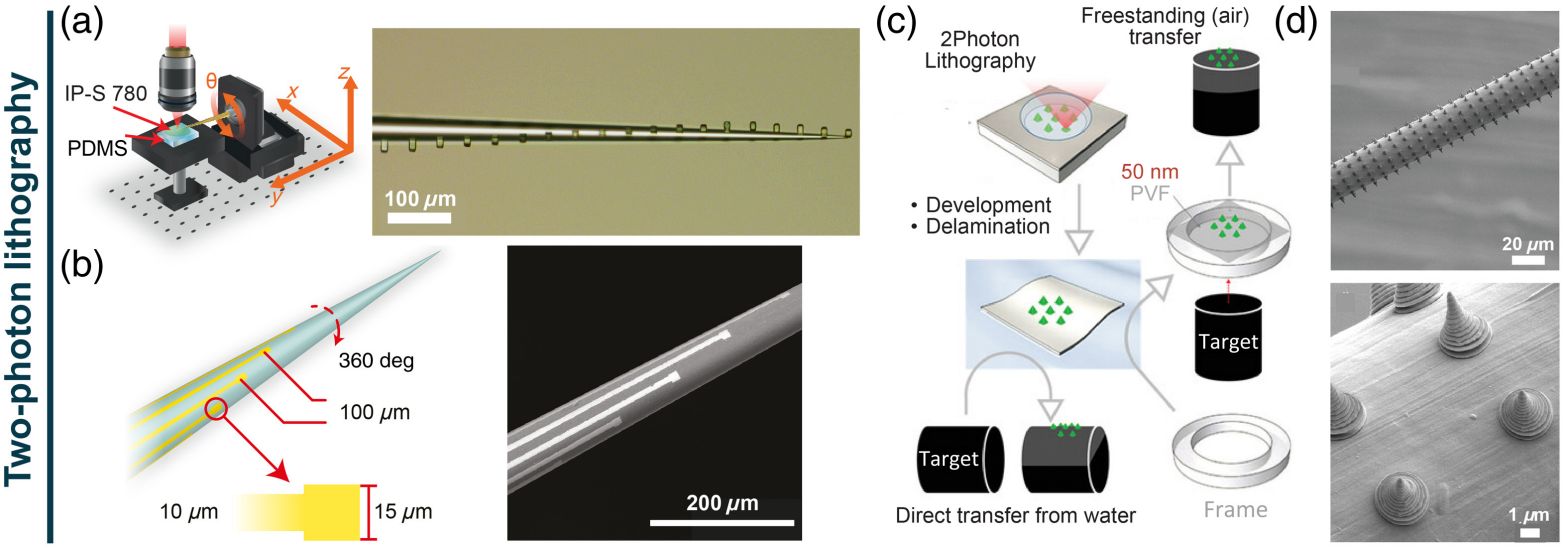
Two-photon lithography. (a) Schematics of the TPL system that allows patterning a TF all around its optical axis and representative optical image. (b) Schematic and scanning electron micrograph of a spiral electrode distribution around a TF. Panels (a) and (b) are reproduced from Ref. 58. (c) Schematics of the fabrication technique that combines TPL and transfer printing. (d) Scanning electron micrograph of a TPL-printed array of 3-μm-size cones wrapped around a ∼20-μm-diameter metal wire. Panels (c) and (d) are adapted from Ref. 59.

A solution that is gaining momentum consists of the two-photon lithography approach (TPL). Thanks to its high spatial resolution, TPL offers the possibility to precisely microstructure a non-planar surface with custom metallic and/or dielectric patterns, in combination with isotropic chemical wet etching routines. TPL has been used to integrate multiple electrodes and different dielectric aperture geometries on a tapered optical fiber [Figs. 3(a)–3(b), which donated the site-selective light delivery—and, potentially, collection—capability^28^^,^^60^^,^^61^ to a multielectrode optrode (*fibertrode*).^58^ As a result of the versatility of this approach, it is not difficult to envision the integration of further microcircuitry elements (resistors, transistors, etc.), paving the way to unconventional multifunctional devices, equipped with micro-resistors for local temperature probing, or OECTs for neurotransmitter release chemical sensing. Furthermore, TPL can also be combined with transfer printing to obtain high-resolution three-dimensional transferable wrappings. As a proof of concept, den Hoed et al.^59^ demonstrated the possibility of transferring an array of complex 3-μm-wide micro-cones all around a 25-μm-diameter tungsten wire using a sacrificial nanometer-thick PVF film, which was successively removed using plasma oxygen [Figs. 3(c)–3(d)]. Despite these promises, TPL remains a serial and, overall, a low-throughput process, especially if compared with the aforementioned MEMS techniques. However, the combination of TPL and holography^62^^,^^63^ can effectively mitigate the low-throughput issue, enabling the parallel fabrication of complex structures, even on multiple fibers at once.

## 4 Discussion and Perspectives

While electrophysiology has historically been considered the gold standard protocol to detect neural signals, the advantages of optogenetic techniques that emerged in the last two decades shifted the paradigm for neuroscience, pushing toward the development of advanced bi-directional optical probes. These two techniques are far from representing the totality of the possibilities that implantable neurophotonics probes could offer, given the great effort of the neurotechnology community to design devices that exploit a growing range of physical phenomena.

The fiber optics platform, compared with competing optical technologies, stands out for several advantages, including cost-effectiveness, ease of use, and bi-directional light delivery and collection. The latter enables unique brain interface methods, such as fiber photometry to monitor neuron state or neurotransmitter release,^64^ as well as to quantify the presence of markers linked to the onset of disease, such as amyloid plaques related to the insurgence of Alzheimer’s disease.^65^ Optical fibers also feature prominently in recent developments in holographic fluorescence imaging endoscopes^39^ and in time-correlated single-photon counting techniques such as fluorescence lifetime photometry (FLiP) to monitor the fluorescence lifetime of specific bioreporters.^66^ Also, the capability to switch the excitation wavelength on the go opens a wide set of experimental possibilities. Infrared light can be employed to perform Raman spectroscopy, to distinguish tissue abnormalities at the molecular level to determine the presence of tumors,^64^^,^^67^ or to monitor biomarkers linked to the insurgence of neurodegenerative pathologies such as Parkinson’s and Alzheimer’s disease.^68^ Other fabrication technologies (i.e., focused ion beam milling,^69^ repeated dewetting^54^), although not discussed in detail in this paper, proved their utility in the integration of plasmonic nano-structures on optical fibers. This allows the exploitation of alternative light-matter interactions such as surface-enhanced Raman spectroscopy (SERS) to increase the Raman response by several orders of magnitude, enabling for instance, the detection of low-concentration neurotransmitters.^70^ Plasmonic nano-structures would also be beneficial for the exploitation of thermoplasmonic effects^71^ to induce localized heating in the brain. Indeed, hyperthermia has been proven to be an effective technique for the treatment of ischemic strokes or certain types of brain tumors.^72^ It has also been proposed to increase the permeability of the blood-brain barrier,^73^ to facilitate the crossing of chemotherapeutic drugs, potentially delivered *in situ* by the same implantable probe, or to thermally trigger capacitance change of the cell membrane.^74^^,^^75^ Alongside it, the capability to locally detect the brain temperature variations in real time with an integrated temperature sensor would be necessary to define a closed-loop heating/sensing system to ensure the positive outcome of these therapies while avoiding cells’ death.

Given the abundance of detection or actuation methods, in some cases already integrated into optical fibers, it is complicated to imagine what a definitive design for a multifunctional implantable probe might be. This wealth is potentially beneficial to the field of neuroscience and gives neurotechnology the opportunity to develop probes driven by research demands. Thus, the point is not so much to envision the ultimate probe as to find the design that best fits the required use. In any case, it is possible to identify certain characteristics that a versatile fiber optics-based multifunctional probe must possess: (i) multisite delivery of light, fully exploiting the large interface area, as in the case of holographic endoscopes, or the extended axial length, as for tapered fibers; (ii) compatibility with optical collection techniques (Raman spectroscopy, fiber photometry, FLiP) for bi-directional interfacing with neural tissue without the need of an additional probe implant; and (iii) high-density electrophysiological recording sites, whether passive (microelectrodes) or active (OECTs), since the electrical channel still remains a feedback of primary importance, even from the perspective of a closed-loop system. Equally important is that the technologies and materials used must be compatible with the possible integration of a multitude of physical channels, as could be temperature sensors, plasmonic structures, or nano-particles to exploit plasmonic light-matter interaction (SERS, hyperthermia), or even microfluidic channels for localized drug delivery. Obviously, integrating even some of these functionalities on a single, minimally invasive device is not an easy task and represents one of the main challenges of the field.

In our opinion, the promising micro-fabrication techniques discussed in this article outline the direction in which the research will head in the coming years although they are often not compatible with each other or require multiple steps that would further complicate the manufacturing and/or its scalability. Fiber drawing remains a very versatile and scalable method, especially for light or drug delivery since multiple cores or channels can be drawn simultaneously. However, optical collection remains a challenge, especially in Raman spectroscopy due to the background signal of the polymers that could completely mask the tissue signal. On the other hand, TPL can be paired with thermal dewetting to combine the excellent optical properties of silica with microelectrodes, temperature sensors, and plasmonic nano-structures; however, its serial nature and the limited space available on the fiber surface puts strong constraints on the scalability. In this context, transfer printing could largely increase the number of electrical elements around the implant, with the tradeoff of less precision in positioning the elements along the probe.

Regardless of the methods and techniques that will take hold in the future, a common challenge for the community will be to face the limited physical space available on implantable optical fibers, setting an upper bound to integration capacity, especially when multiple features should coexist. A bigger fiber cross-section may facilitate the integration of multiple functionalities, albeit at the expense of the overall invasiveness of the implant. Nevertheless, in such a fervent and fast-moving research scenario,^76^^–^^78^ it is easy to imagine that these developments could be combined in innovative hybrid approaches that would allow the advantages and disadvantages of each technique to counterbalance each other.

## Data Availability

Data sharing is not applicable.
